# nextMONARCH Phase 2 randomized clinical trial: overall survival analysis of abemaciclib monotherapy or in combination with tamoxifen in patients with endocrine-refractory HR + , HER2– metastatic breast cancer

**DOI:** 10.1007/s10549-022-06662-9

**Published:** 2022-07-12

**Authors:** Erika Hamilton, Javier Cortes, Ozgur Ozyilkan, Shin-Cheh Chen, Katarina Petrakova, Aleksey Manikhas, Guy Jerusalem, Roberto Hegg, Jens Huober, Wei Zhang, Yanyun Chen, Miguel Martin

**Affiliations:** 1 Breast and Gynecologic Research Program, Sarah Cannon Research Institute/Tennessee Oncology PLLC, 250 25th Ave North, Suite 200, Nashville, TN 37203 USA; 2 International Breast Cancer Center (IBCC), Quironsalud Group, Barcelona, Spain; 3 Faculty of Biomedical and Health Sciences, Department of Medicine, Universidad Europea de Madrid, Madrid, Spain; 4 Department of Medical Oncology, Baskent University, Adana, Turkey; 5 Chang Gung Memorial Hospital, Chang Gung University, Linkou, Taoyuan City, Taiwan; 6 Masarykuv Onkologický Ustav, Brno, Czech Republic; 7 City Clinical Oncology Center, St. Petersburg, Russia; 8 Centre Hospitalier Universitaire, Liège, and Liège University, Liège, Belgium; 9 Centro de Referência da Saúde da Mulher, Hospital Pérola Byington/FMUSP, São Paulo, Brazil; 10 Breast Center, University of Ulm, Ulm, Germany; 11 Breast Center, Cantonal Hospital, St Gallen, Switzerland; 12 Eli Lilly and Company, Indianapolis, IN USA; 13 Instituto de Investigación Sanitaria Gregorio Marañón, Universidad Complutense, CIBERONC, GEICAM, Madrid, Spain

**Keywords:** Cyclin-dependent kinase 4 and 6, Endocrine therapy, HER2-negative, Hormone receptor-positive, MBC, Overall survival

## Abstract

**Purpose:**

Resistance to endocrine therapy poses a major clinical challenge for patients with hormone receptor-positive (HR +), human epidermal growth factor receptor 2-negative (HER2–) metastatic breast cancer (MBC). We present the preplanned 24-month final overall survival (OS) results, alongside updated progression-free survival (PFS), and objective response rate (ORR) results.

**Methods:**

nextMONARCH is an open-label, controlled, randomized, Phase 2 study of abemaciclib alone or in combination with tamoxifen in women with endocrine-refractory HR + , HER2– MBC previously treated with chemotherapy. Patients were randomized 1:1:1 to: abemaciclib 150 mg and tamoxifen 20 mg (A + T), abemaciclib 150 mg (A-150), or abemaciclib 200 mg and prophylactic loperamide (A-200). OS was the main prespecified secondary endpoint. PFS, ORR, and safety at 24 months were compared to previously reported primary analysis results.

**Results:**

Of the 234 patients enrolled, 12 were receiving study treatment at data cutoff (28Jun2019). Median follow-up was 27.2 months. Median OS was 24.2 months in the A + T arm, 20.8 months in A-150, and 17.0 months in A-200 (A + T versus A-200: HR 0.62; 95%CI [0.40, 0.97], *P* = 0.03 and A-150 versus A-200: HR 0.96; 95%CI [0.64, 1.44], *P* = 0.83). PFS and ORR results at 24 months were consistent with the primary analysis. The safety profile corresponded with previous reports.

**Conclusion:**

The addition of tamoxifen to abemaciclib demonstrated greater OS benefit than monotherapy. This study confirmed the single-agent activity of abemaciclib in heavily pretreated women with endocrine-refractory HR + , HER2– MBC, as well as the previously reported primary PFS and ORR results, with no new safety signals observed.

**Trial Registration** ClinicalTrials.gov Identifier: NCT02747004.

**Supplementary Information:**

The online version contains supplementary material available at 10.1007/s10549-022-06662-9.

## Introduction

Hormone receptor-positive (HR +), human epidermal growth factor 2-negative (HER2–) metastatic breast cancer (MBC) represents approximately 70% of all MBCs. Endocrine therapy (ET) is the mainstay of treatment for patients with MBC; however, despite the benefit of ET in the advanced setting, patients ultimately develop endocrine resistance [1, 2]. Patients with MBC have a poor prognosis, with a median overall survival (OS) of approximately 3 years and a 5-year survival rate of approximately 25% [3]. Resistance to ET poses a major clinical challenge, and as a result, there is a medical need to improve current therapeutic strategies to prolong patient survival [4].

Inhibition of cyclin-dependent kinase 4 and 6 (CDK4 and 6) has proven to be effective in attenuating ET resistance, and several studies investigating the efficacy of CDK4 and 6 inhibitors in combination with ET have shown statistically significant improvements in efficacy outcomes when compared to ET alone [5–8]. As a result of the compelling PFS and quality of life benefits, the combination of CDK4 and 6 inhibitor with ET is now recognized as a new standard of care for patients with HR + , HER2– advanced or MBC [3].

Abemaciclib, an oral selective inhibitor of CDK4 and 6 administered on a continuous schedule, has demonstrated statistically and clinically meaningful efficacy in patients with HR + , HER2– advanced or MBC as monotherapy and in combination with ET. In the Phase 2 MONARCH 1 trial, abemaciclib monotherapy (200 mg twice per day [BID]), demonstrated an objective response rate (ORR) of 19.7% (95% CI [13.3, 27.5]; 15% not excluded) with a median progression-free survival (PFS) of 6.0 months and median OS of 17.7 months [9] for women with HR + , HER2– MBC following prior ET and chemotherapy. As a result of the MONARCH 1 trial, abemaciclib is the only drug in its class approved by the U.S. Food and Drug Administration as a monotherapy for endocrine-refractory MBC. Abemaciclib has also received global approval for the treatment of advanced or MBC in combination with fulvestrant (MONARCH 2 trial) or in combination with a nonsteroidal aromatase inhibitor (AI) (MONARCH 3 trial) [4, 7]. More recently, abemaciclib became the first CDK4 and 6 inhibitor in its class approved for adjuvant treatment of HR + , HER2–, node-positive, early breast cancer [10].

The nextMONARCH trial (NCT02747004) was a Phase 2, randomized, open-label study of abemaciclib as a monotherapy or a combination therapy with tamoxifen in women with previously treated HR + , HER2– MBC. The trial compared the efficacy of (a) abemaciclib (150 mg BID) plus tamoxifen (A + T) and (b) single-agent abemaciclib (150 mg BID) (A-150), relative to the recommended (c) single-agent dose of abemaciclib (200 mg BID) combined with prophylactic loperamide (A-200) in this patient population.

Primary analysis from this trial showed that the addition of tamoxifen to abemaciclib therapy resulted in numerically increased PFS in A + T compared to A-200 (median PFS = 9.1 months and 7.4 months, respectively; hazard ratio [HR] 0.815, *P* = 0.293). PFS in A-150 was comparable to that in A-200 (median PFS = 6.5 months and 7.4 months, respectively; HR 1.045, *P* = 0.811). The investigator assessed unconfirmed ORR was 34.6% (A + T), 24.1% (A-150) and 32.5% (A-200). Occurrence of treatment related diarrhea was well controlled by a combination of dose adjustments and the anti-diarrheal medication loperamide [11]. Herein we present a prespecified final 24-month follow-up analysis of OS after the last patient entered treatment, and updated PFS and ORR results.

## Methods

### Study design and patients

The nextMonarch trial was a Phase 2 randomized, open-label study of abemaciclib as a monotherapy or in combination with tamoxifen for the treatment of HR + , HER2–, MBC in women aged ≥ 18 years. Patients were enrolled at 60 sites in 14 different regions/countries. All patients enrolled had disease progression during or after ET and had received ≥ 2 prior chemotherapy regimens, at least 1 but no more than 2 of which were administered in the metastatic setting. Detailed inclusion and exclusion criteria were previously described [11]. All patients provided written informed consent before enrollment. The study was approved by the local ethical and institutional review boards for all participating sites and was conducted according to the Declaration of Helsinki’s Good Clinical Practice. This study followed the Consolidated Standards of Reporting Trials (CONSORT) reporting guideline.

### Randomization and treatment

Eligible patients were randomly assigned in a 1:1:1 ratio using an interactive web- response system (IWRS) to receive a combination therapy of abemaciclib (150 mg BID) with tamoxifen (20 mg daily) (A + T), abemaciclib monotherapy (150 mg BID) (A-150), or abemaciclib monotherapy (200 mg BID) with prophylactic loperamide (2 mg daily) (A-200). Randomization was stratified by the presence of liver metastases (yes *vs* no) and prior tamoxifen therapy in the advanced/metastatic setting (yes *vs* no). The IWRS used randomization factors to assign study treatment to each patient. All drugs were orally administered in a 28-day cycle. Patient stratification factors, as well as treatment dosing and adjustment details have previously been described [11]. Study treatment continued until disease progression, unacceptable toxicity, or death.

### Efficacy and safety assessment

Tumors were assessed by computed tomography (CT) or magnetic resonance imaging (MRI) according to RECIST version 1.1, within 4 weeks before randomization and at every other cycle (8 weeks) thereafter. Safety evaluations at all patient visits included vital signs, physical examination, clinical laboratory and adverse event (AE) assessments using the National Cancer Institute Common Terminology Criteria for Adverse Events version 4.0 and coded using the Medical Dictionary for Regulatory Activities (MedDRA) version 18.1 (or higher).

### Endpoints

The primary endpoint was investigator-assessed PFS, measured from the date of randomization to the date of objective disease progression or death from any cause, whichever was earlier. The secondary endpoint, OS was measured from the date of randomization to the date of death from any cause or to the date of last patient contact (censoring date), whichever was earlier. Other secondary endpoints included ORR (percentage of patients with complete response [CR] or partial response [PR]), duration of response (DoR, time from the date of first evidence of CR or PR to disease progression or death from any cause, whichever was earlier), and clinical benefit rate (CBR, the percentage of patients with CR, PR, or stable disease for ≥ 6 months).

### Statistical analysis

The nextMonarch study final analysis compared OS (secondary endpoint) of patients treated with abemaciclib in combination with tamoxifen (A + T) and abemaciclib monotherapy (A-150 and A-200) and analyzed up-to-date PFS and ORR data. Efficacy analyses were conducted on the intent-to-treat (ITT) population: the primary statistical analysis tested the superiority of PFS in A + T compared to A-200, while an informal non-inferiority analysis compared PFS between A-150 and A-200. Power calculations for PFS analysis were previously described [11]. A stratified Cox regression model was used to estimate the HR between treatment arms. The secondary efficacy analysis evaluated ORR, DoR, and OS of each arm. The planned final OS analysis was performed 24 months after the last patient entered treatment. Subgroup analyses of OS were assessed on prespecified patient subgroups as specified in the study protocol. For the subgroup analysis, HR between treatment arms and 95% CI (Wald) were estimated from unstratified Cox model. The safety population included all enrolled patients that received at least one dose of treatment.

## Results

### Patients and treatment

**Fig. 1:**
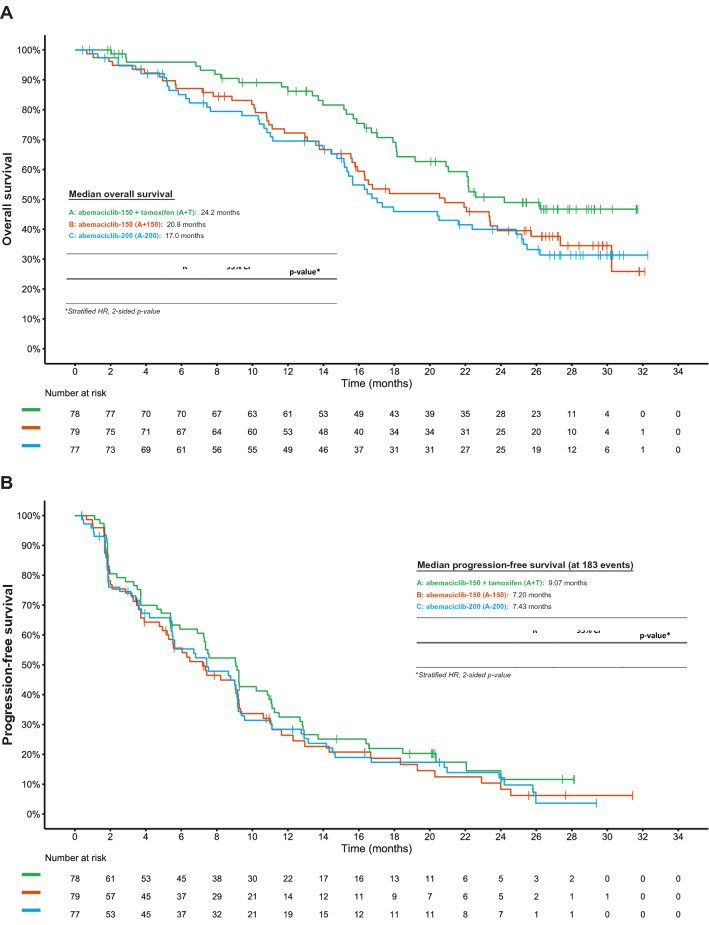
Kaplan–Meier curves of survival in the intent-to-treat population, **a** overall survival at 24 months and **b** progression free survival at 183 events

From September 2016 to June 2017, a total of 234 patients were randomly assigned to A + T (*n* = 78), A-150 (*n* = 79), or A-200 (*n* = 77) (Fig. 1). As previously described in the primary analysis, baseline patient and disease characteristics were well balanced among the treatment arms (Supplemental Table 1) [11]. At the time of the data cut-off (28June2019) 12 patients (5.1%) were still receiving treatment (6 patients in the A + T arm [7.7%]; 3 patients in the A-150 [3.8%] and A-200 [3.9%] arms) (Fig. 1). By the data cut-off date, 222 patients (94.9%) had discontinued from treatment; and primary reason for treatment discontinuation was progressive disease (179 patients [76.5%]).

**Table 1:** Best overall response by investigator assessment and confirmed

*n* (%)	A + T arm		A-150 arm		A-200 arm		Odds ratio^c^		*P* value^d^	
	(*n* = 78)		(*n* = 79)		(*n* = 77)					
	*n* (%)	95% CI^b^	*n* (%)	95% CI^b^	*n* (%)	95% CI^b^	A + T *vs*	A-150 *vs*	A + T *vs*	A-150 *vs*
							A-200	A-200	A-200	A-200
Best overall response^a^										
CR	1 (1.3)	0.0–3.8	1 (1.3)	0.0–3.7	0 (0)	N/A	–	–	–	–
PR	26 (33.3)	22.9–43.8	18 (22.8)	13.5–32.0	26 (33.8)	23.2–44.3	–	–	–	–
SD	35 (44.9)	33.8–55.9	36 (45.6)	34.6–56.6	27 (35.1)	24.4–45.7	–	–	–	–
SD for ≥ 6 months	21 (26.9)	17.1–36.8	20 (25.3)	15.7–34.9	14 (18.2)	9.6–26.8	–	–	–	–
PD	15 (19.2)	10.5–28.0	15 (19.0)	10.3–27.6	17 (22.1)	12.8–31.3	–	–	–	–
Objective PD	15 (19.2)	10.5–28.0	15 (19.0)	10.3–27.6	17 (22.1)	12.8–31.3	–	–	–	–
Non evaluable	1 (1.3)	0.0–3.8	9 (11.4)	4.4–18.4	7 (9.1)	2.7–15.5	–	–	–	–
ORR (CR + PR)	27 (34.6)	24.1–45.2	19 (24.1)	14.6–33.5	26 (33.8)	23.2–44.3	0.9	0.6	0.8719	0.1195
DCR (CR + PR + SD)	62 (79.5)	70.5–88.4	55 (69.6)	59.5–79.8	53 (68.8)	58.5–79.2	1.6	1	0.23	0.9306
CBR (CR + PR + persistent SD^b)^	48 (61.5)	50.7–72.3	39 (49.4)	38.3–60.4	40 (51.9)	40.8–63.1	1.3	0.8	0.3825	0.6172
PFS, median mos	9.07		7.2		7.43		HR (95% Cl), *P*-value			
							A + T *vs* A-200 = 0.81 (0.56, 1.16), 0.2493			
							A-150 *vs* A200 = 1.06 (0.74, 1.53), 0.7400			
OS, median mos	24.2		20.8		17		HR (95% Cl), *P*-value			
							A + T *vs* A-200 = 0.62 (0.40, 0.97), 0.0341			
							A-150 *vs* A200 = 0.96 (0.64, 1.44), 0.8321			

*A* + *T* abemaciclib 150 mg plus tamoxifen, *A-150* abemaciclib 150 mg, *A-200* abemaciclib 200 mg plus prophylactic loperamide, *CI* confidence interval, *CR* complete response, *N* total number of patients randomized, *n* number of patients in category, *OS* overall survival, *PD* progressive disease, *PFS*, progression-free survival, *PR* partial response, *SD* stable disease

^a^Investigator-assessed response according to Response Evaluation Criteria In Solid Tumors (RECIST), version 1.1

^b^Based on normal approximation

^c^Stratified by the presence of liver metastases and prior tamoxifen use for locally advanced/metastatic breast cancer

^d^*P*-value is calculated by Asymptotic Cochran-Mantel–Haenszel test stratified by the presence of liver metastases and prior tamoxifen use for locally advanced/metastatic breast cancer

### Efficacy

**Fig. 2:**
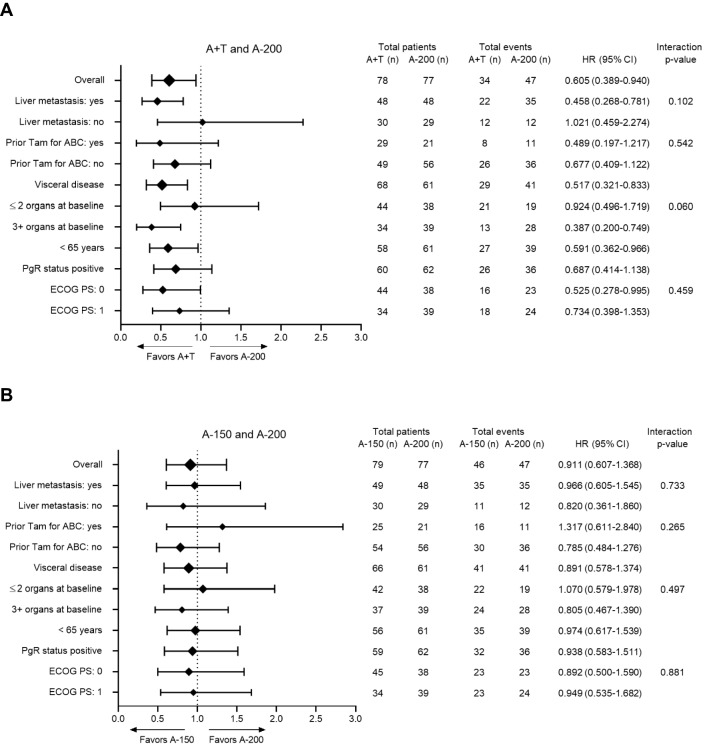
Sub-group analysis of overall survival in the intent-to-treat population. Overall survival unstratified hazard ratios (HR) and 95% confidence intervals are shown with diamond size proportional to the number of patients in each sub-group. Factor levels with < 33% of randomized patients were omitted from the analysis except for randomization stratification factors, notably presence of liver metastasis and previous use of tamoxifen in the advanced or metastatic setting. *ABC* advanced breast cancer, *A* + *T* abemaciclib 150 mg plus tamoxifen, *A-150* abemaciclib 150 mg, *A-200* abemaciclib 200 mg plus prophylactic loperamide, *CI* confidence internal, *HR* hazard ratio, *n* number of subjects in the subgroup, *PS* performance status, *T* tamoxifen

**Fig. 3:**
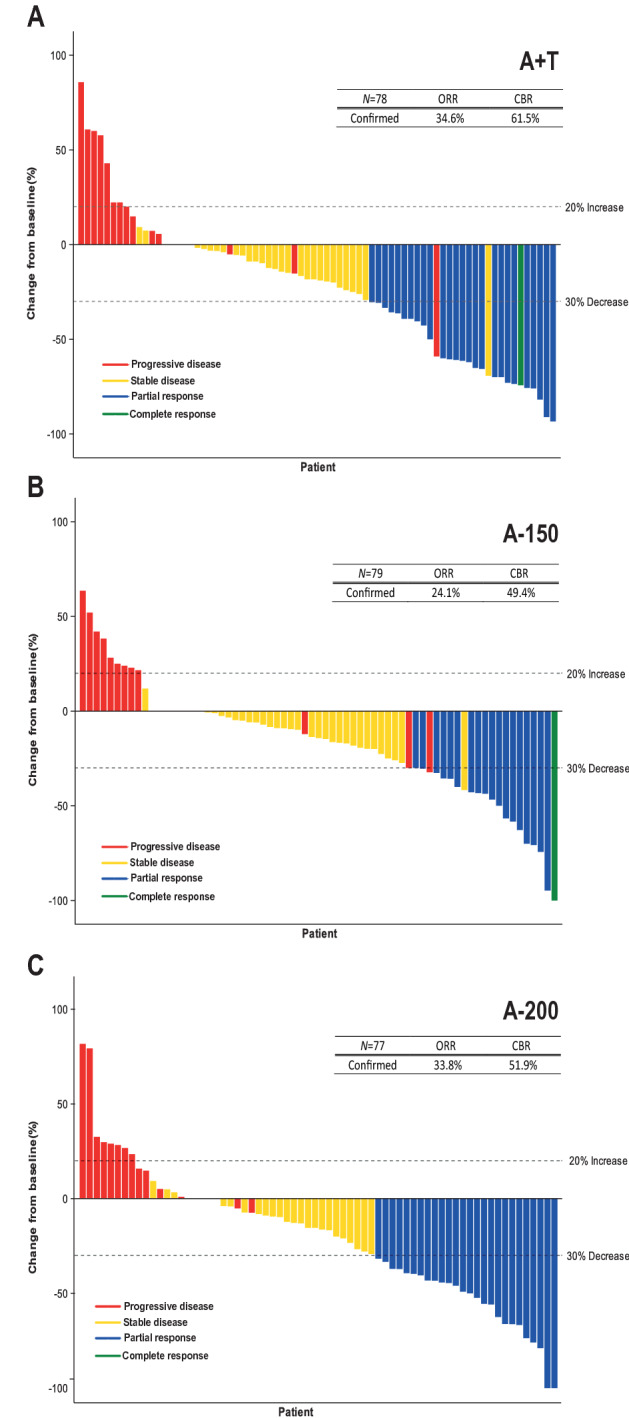
Best percentage change in tumor size from baseline, each bar representing individual patients with measureable disease. *A* + *T* abemaciclib 150 mg plus tamoxifen, *A-150* abemaciclib 150 mg, *A-200* abemaciclib 200 mg plus prophylactic loperamide, *CBR* clinical benefit rate, *N* total number of patients randomized, *ORR* objective response rate

The A + T arm demonstrated an extended median OS (24.2 months) when compared to the A-200 arm (17.0 months, HR = 0.62; 95% CI [0.40, 0.97]; *P* = 0.0341) (Fig. 2A). Median OS for the A-150 arm (20.8 months) was similar compared to that of the A-200 arm (17.0 months, HR 0.96; 95% CI [0.64, 1.44]; *P* = 0.8321). The median length of follow-up was 27.2 months. As of the final analysis, the estimate of the 24-month OS probability was 50.8% (95% CI [37.9%, 62.3%]) for A + T, 39.5% (95% CI [28.0%, 50.8%]) for A-150, and 40.0% (95% CI [28.4%, 51.2%]) for A-200. In addition, predefined subgroup analyses of OS were performed for each of the baseline characteristics: nature of visceral disease; number of involved organ sites; patient age; progesterone receptor status; and Eastern Cooperative Oncology Group performance status. No statistically significant treatment-by-subgroup interactions were observed in the subgroup OS analyses for A + T arm *vs* A-200 (Fig. 3A) or A-200 *vs* A-150 (Fig. 3B).

At the final analysis, 183 PFS events had occurred of which 61 were in the A + T arm, 60 in the A-150 arm, and 62 in the A-200 arm. A numerically longer PFS was observed for the A + T arm (9.1 months) when compared to the A-200 arm (7.4 months) (HR 0.81; 95% CI [0.56, 1.16]; *P* = 0.2493) (Fig. 2B). The median PFS for A-150 (7.2 months) and A-200 (7.4 months) were comparable (HR 1.06, 95% CI [0.74, 1.53]; *P* = 0.7400).

In line with the primary analysis report, the investigator-assessed ORR was 34.6% (95% CI [24.1%, 45.2%]) for A + T, 24.1% (95% CI [14.6%, 33.5%])for A-150, and 33.8% (95% CI [23.2%, 44.3%]) for A-200 (A + T *vs* A-200: *P* = 0.8719, A-150 *vs* A-200: *P* = 0.1195) (Table 1). One additional patient in the A + T arm achieved a CR (one CR was previously observed for A-150). Again, all treatment arms showed a durable response, with a median DoR of 7.40 months (A + T), 8.40 months (A-150), and 8.45 months (A-200) (Supplemental Table 3). Similar to the primary analysis report, tumor shrinkage was observed in over 70% of patients across all three arms; 39.2% (A + T), 31.9% (A-150), and 38.2% (A-200) had a maximum decrease of baseline lesion size ≥ 30% (Fig. 3). Finally, clinical benefit rate (CBR) mirrored the primary analysis results; 61.5% in A + T, 49.4% in A-150, and 51.9% in A-200 (A + T vs A-200: *P* = 0.3825, A-150 vs A-200: *P* = 0.6172).

### Safety

Abemaciclib dose reductions and omissions due to AEs were similar to those reported in the primary analysis and are presented in Supplemental Table 2. The incidence of treatment-emergent adverse events (TEAEs) was largely consistent with the primary analysis results and with the findings from previously reported abemaciclib breast cancer studies; 226 patients experienced one or more TEAEs (96.6%) (Table 2). Overall, the most frequently reported TEAEs of any grade, regardless of causality, in ≥ 25% of patients included diarrhea (61.1%); neutropenia (49.6%); anemia (40.6%); nausea (36.3%); leukopenia (30.8%); fatigue (29.9%); and abdominal pain (27.4%). The occurrence ≥ grade 3 events were greatest for neutropenia (30.3%), followed by leukopenia (11.5%) and anemia (11.1%). The A + T and A-200 arms had a higher incidence of grade ≥ 3 AST increased than the A-150 arm.

**Table 2:** Treatment-emergent adverse events occurring in ≥ 15% of the safety population, regardless of causality

Grade (> 15% occurrence); *n* (%)	A + T arm (*n* = 78)		A-150 arm (*n* = 79)		A-200 arm (*n* = 77)	
	Any grade	≥ 3 Grade	Any grade	≥ 3 Grade	Any grade	≥ 3 Grade
Any adverse event^a^	73 (93.6)	43 (55.1)	77 (97.5)	42 (53.2)	76 (98.7)	57 (74.0)
Diarrhea	42 (53.8)	1 (1.3)	53 (67.1)	3 (3.8)	48 (62.3)	7 (9.1)^#^
Neutropenia	33 (42.3)	18 (23.1)	43 (54.4)	24 (30.4)	40 (51.9)	29 (37.7)
Anemia	34 (43.6)	11 (14.1)	27 (34.2)	6 (7.6)	34 (44.2)	9 (11.7)
Nausea	25 (32.1)	2 (2.6)	26 (32.9)	2 (2.5)	34 (44.2)	2 (2.6)
Leukopenia	22 (28.2)	8 (10.3)	28 (35.4)	10 (12.7)	22 (28.6)	9 (11.7)
Fatigue	25 (32.1)	3 (3.8)	21 (26.6)	2 (2.5)	24 (31.2)	5 (6.5)
Abdominal pain	21 (26.9)	0 (0.0)	18 (22.8)	1 (1.3)	25 (32.5)	0 (0.0)
Thrombocytopenia	15 (19.2)	3 (3.8)	13 (16.5)	4 (5.1)	28 (36.4)	5 (6.5)
Vomiting	14 (17.9)	2 (2.6)	20 (25.3)	3 (3.8)	20 (26.0)	4 (5.2)
Decreased appetite	20 (25.6)	4 (5.1)	12 (15.2)	1 (1.3)	17 (22.1)	2 (2.6)
Constipation	11 (14.1)	0 (0.0)	9 (11.4)	0 (0.0)	26 (33.8)	1 (1.3)
Increased blood creatinine	14 (17.9)	1 (1.3)	9 (11.4)	0 (0.0)	8 (10.4)	0 (0.0)
Muscular weakness	13 (16.7)	2 (2.6)	12 (15.2)	1 (1.3)	7 (9.1)	2 (2.6)
Dyspnea	9 (11.5)	2 (2.6)	14 (17.7)	3 (3.8)	6 (7.8)	0 (0.0)

TEAE of special interest:	Any grade	Grade 3/4	Any grade	Grade 3/4	Any grade	Grade 3/4
Venous thromboembolism	8 (10.3)	3 (3.8)	3 (3.8)	2 (2.5)	3 (3.9)	1 (1.3)
Pneumonitis	0 (0.0)	0 (0.0)	1 (1.3)	0 (0.0)	2 (2.6)	1 (1.3)
AST increased	8 (10.3)	2 (2.6)	4 (5.1)	0 (0.0)	8 (10.4)	2 (2.6)
ALT increased	6 (7.7)	2 (2.6)	4 (5.1)	2 (2.5)	6 (7.8)	3 (3.9)

*A* + *T* abemaciclib 150 mg + tamoxifen, *A-150* abemaciclib 150 mg, *A-200* abemaciclib 200 mg + prophylactic loperamide, *TEAE* treatment-emergent adverse event, *AST* aspartate aminotransferase, *ALT* alanine aminotransferase, *N* safety population, *n* number of patients affected

^a^6 patients (2 in each arm) also had grade 5 events (2 = disseminated intravascular coagulation, [A + T and A-200], 2 = cardiac arrest [A + T and A-150], 1 = multiorgan disfunction syndrome [A-150], 1 = aspiration [A-200])

^#^Since the primary disclosure [11], no new diarrhea cases were reported, but one patient with G2 diarrhea advanced to G3 diarrhea in cycle 17

Since the 12-month analysis, no additional diarrhea events were reported. When compared to the A-150 and A-200 arms, patients in the A + T arm required fewer dose adjustments due to diarrhea and experienced fewer any grade and grade ≥ 3 diarrhea events. The TEAEs of embolism were reported for 1 additional patient in A + T (8 patients [10.3%]). No additional patients reported TEAEs of embolism in A-150 and A-200 (A-150: 3 patients [3.9%]; and A-200: 3 patients [3.9%]) (Table 2). At 24-month analysis, TEAE of neutropenia (any grade) was reported for 1 additional patient in A + T arm (33 patients [42.3%]; grade ≥ 3: 18 patients [23.1%]), 3 additional patients in A-150 (43 patients [54.4%]; grade ≥ 3: 24 patients [30.4%]), and no additional patient reported TEAE of neutropenia in A-200 (40 patients [51.9%; grade ≥ 3: 29 patients [37.7%]). No additional events of pneumonitis or transaminase enzyme elevations (aspartate aminotransferase [AST] or alanine aminotransferase [ALT]) have been reported since the 12-month analysis. There were no additional deaths reported due to AEs while on study treatment or within 30 days of discontinuation at the 24-month final analysis.

## Discussion

We present final analysis results of an open-label Phase 2 trial of abemaciclib plus tamoxifen or abemaciclib alone in women with previously treated HR + , HER2 − , MBC whose disease had progressed on or after endocrine therapy and chemotherapy. nextMONARCH data reconfirms the antitumor activity of abemaciclib as monotherapy (200 mg BID) in pre-treated patients with HR + , HER2– MBC in line with MONARCH 1 results, which had a similar patient profile [9, 11]. In the previously reported primary analysis, abemaciclib in combination with tamoxifen resulted in a numerically longer but not statistically significant median PFS in the A + T arm compared to the A-200 arm (1.7 months greater). At the time of the primary analysis of PFS, overall survival data were immature.

Although this study was not designed to test OS superiority, the A + T arm had a longer OS compared to the A-200 arm (24.2 and 17.0 months, respectively, HR 0.62, *P* < 0.05) in the final analysis. A-150 OS (20.8 months) was similar to A-200 (17.0 months) (HR 0.956; [CI 95% 0.635, 1.438], *P* = 0.8321). The results of the 24-month median PFS analysis were consistent with the findings of the primary analysis. This data is in line with previous reports that inhibiting both the estrogen receptor pathway and the cell cycle is more effective than inhibiting the cell cycle alone [12, 13].

Improving OS is considered the most important therapeutic goal in advanced breast cancer and is a universally accepted direct measure of clinical benefit. However, before mortality is reached, the relationship between drug effectiveness and patient survival can be confounded by a range of factors including post-discontinuation therapy, and this may be why we observe a significant difference in OS and not PFS in this study. PFS is reflective of tumor growth and directly measures the impact of study intervention without the confounder of post study therapy, and this is why PFS was chosen as the primary endpoint for this study.

The ORR, DCR, CBR, and PFS were similar between the 12- and 24-month analyses. Median OS and PFS reported in the final analysis for A-200 were also comparable to those documented in the MONARCH 1 trial (17 months *vs* 17.7 months; 7.43 months *vs* 6.0 months, respectively). In addition to ORR improvements, CBR improved for A-200 in this study when compared to Monarch 1 (ORR: 24.1 *vs* 19.7; CBR: 49.4 *vs* 42.4, respectively).

Though the investigator-assessed ORR was numerically higher in the A-200 arm, the reported median OS for A-150 (20.8 months) compared well to A-200 (17.0 months) (HR 0.96; 95%CI [0.64, 1.44], *P* = 0.83). Median PFS in A-150 was also similar to that observed in A-200 (7.20 months and 7.43 months, respectively).

From a safety perspective, TEAEs of abemaciclib treatment were consistent with those previously reported and within the known safety parameters. Diarrhea was generally low grade, and typically occurred early in the course of treatment. When compared to A + T or A-150 arms, the addition of prophylactic loperamide to the A-200 arm did not result in a reduction in the incidence of any grade and ≥ 3 grade diarrhea events. However, the incidence of diarrhea across arms was lower than in MONARCH 1 (all occurrences: 62.3% compared to 90.2%, respectively) and severity was lower (Grade 3 events: 9.1% compared to 19.7%), indicating that the diarrhea management plan, which was implemented at a later stage of Monarch 1 and in all subsequent studies, not necessarily prophylactic loperamide, was effective in preventing and managing diarrhea, making abemaciclib more tolerable [9].

Neutropenia levels (≥ grade 3 events ranging from 23.1% to 37.7%) were consistent with previous abemaciclib studies [10]. Although increased AST and ALT levels were reported in all three treatment arms, the occurrence of ≥ 3 grade events were aligned with previous abemaciclib studies; the number of all grade events were considerably lower in comparison to MONARCH 1.

The prevalence of venous thromboembolisms (VTEs) is noteworthy, particularly considering the known risk associated with tamoxifen therapy[14]. A total of 18 patients (20 events) are reported here, higher than reported in the primary analysis and likely explained by the longer follow-up. As expected, incidence was higher in the A + T arm (10.3%) compared to A-150 (3.8%) and A-200 (3.9%). Most of the events were managed with anticoagulant therapy, with only one patient (A + T arm) having to discontinue treatment.

Since the 12-month analysis, no additional events of pneumonitis have been reported. In total, pneumonitis was reported for 3 patients, 2 in the A-200 arm and 1 in the A-150 arm and were resolved following dose omission or dose reduction.

Integration of these data into clinical practice should warrant several important considerations. First, though patients enrolled on this study were heavily pre-treated, they were naïve to CDK 4 and 6 therapy; therefore, this study does not address the activity of A or A + T in patients who have received prior CDK 4 and 6 inhibitor therapy. However, our findings suggest that abemaciclib and tamoxifen might potentially be an effective later line treatment for patients who have not received prior CDK 4 and 6 inhibitor therapy and for those who are intolerant to other endocrine therapy backbones. Additionally, patients who are candidates for therapy should be counseled concerning the increased risks of thrombotic events associated with A + T in addition to the improvement in OS offered by this regimen.

## Conclusion

Our final analysis reconfirmed the single-agent activity of abemaciclib in heavily pretreated HR + , HER2– MBC. The addition of tamoxifen to abemaciclib resulted in longer OS when compared to abemaciclib monotherapy and may potentially be an effective later line treatment for patients who are CDK 4 and 6 inhibitor therapy naïve or are intolerant to other endocrine therapy backbones.

## Supplementary Information

Below is the link to the electronic supplementary material.

Supplementary file1 (DOCX 728 kb)

## Data Availability

Eli Lilly and Company provides access to all individual participant data collected during the trial, after anonymization, with the exception of pharmacokinetic or genetic data. Data are available to request 6 months after the indication studied has been approved in the US and EU and after primary publication acceptance, whichever is later. No expiration date of data requests is currently set once data are made available. Access is provided after a proposal has been approved by an independent review committee identified for this purpose and after receipt of a signed data sharing agreement. Data and documents, including the study protocol, statistical analysis plan, clinical study report, blank or annotated case report forms, will be provided in a secure data sharing environment. For details on submitting a request, see the instructions provided at www.vivli.org.
