# Supplementary material for: nextMONARCH Phase 2 randomized clinical trial: overall survival analysis of abemaciclib monotherapy or in combination with tamoxifen in patients with endocrine-refractory HR + , HER2– metastatic breast cancer
Source: Breast Cancer Res Treat. 2022 Jul 12;195(1):55–64. doi: 10.1007/s10549-022-06662-9 (PMC9338008; doi:10.1007/s10549-022-06662-9)
Supplement: Supplementary file 1 — Supplementary file1 (DOCX 728 kb) [file 10549_2022_6662_MOESM1_ESM.docx]

**Supplemental Material**

**Supplemental Figure 1. (Online only) CONSORT Diagram Showing Patient Inclusion and Exclusion and Outcomes**


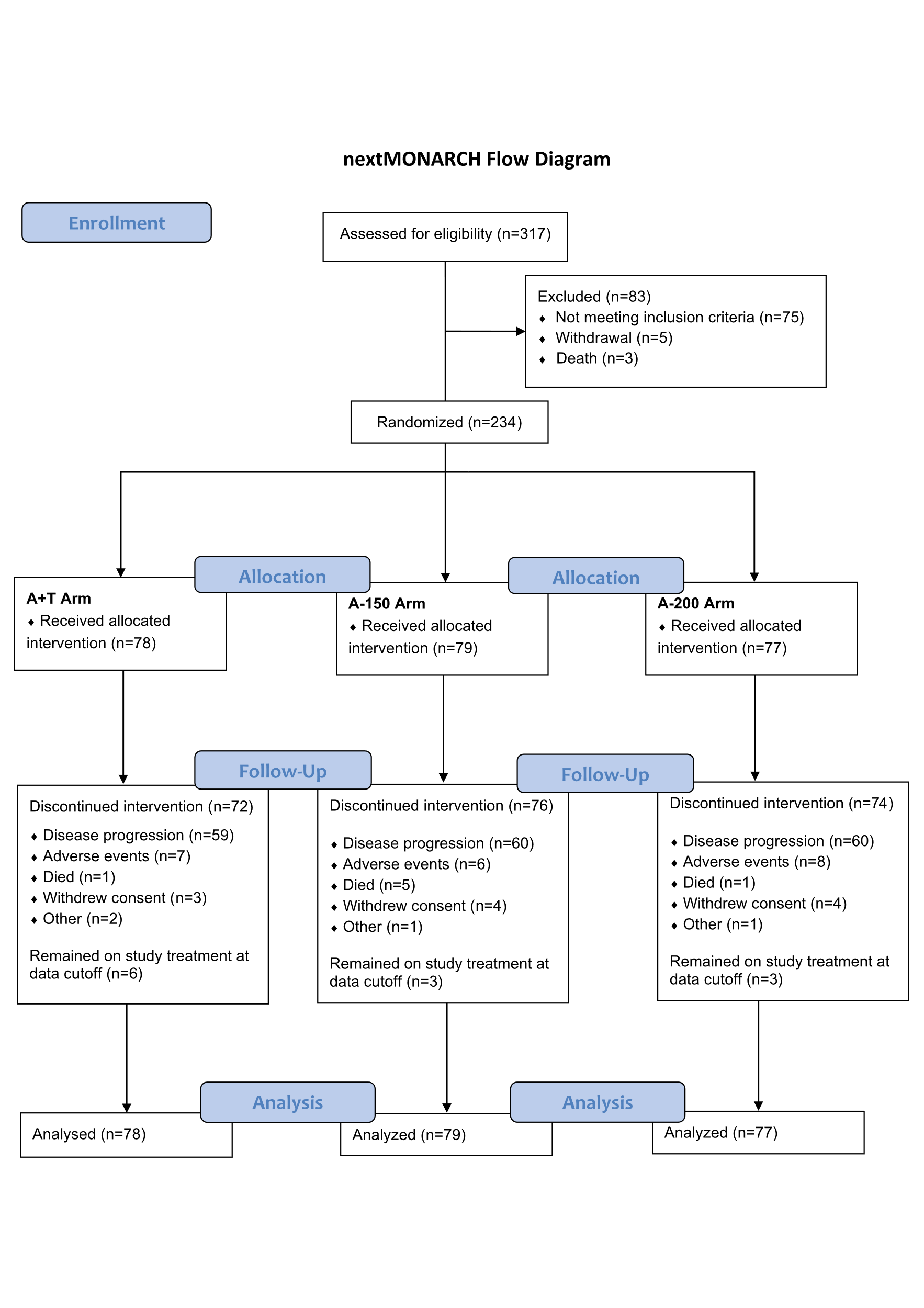


**Supplemental Table 1 (Online only). Patient and Disease Baseline Characteristics**

| **Characteristics** | **A+T Arm**  ***(*n=78)** | **A-150 Arm**  **(n=79)** | **A-200 Arm**  **(n=77)** |
| --- | --- | --- | --- |
| **Age, years, median (range)** | 53 (32-77) | 56 (32–81) | 56 (35-77) |
| **Race^a^, *n* (%)** |  |  |  |
| White | 63 (81.8) | 64 (85.3) | 60 (78.9) |
| Asian | 8 (10.4) | 6 (8.0) | 10 (13.2) |
| Other^b^/missing | 7 (9.0) | 9 (11.4) | 7 (9.1) |
| **Liver metastases** |  |  |  |
| Yes | 48 (61.5) | 49 (62.0) | 48 (62.3) |
| No | 30 (38.5) | 30 (38.0) | 29 (37.7) |
| **Previous Tamoxifen for ABC** |  |  |  |
| Yes | 29 (37.2) | 25 (31.6) | 21 (27.3) |
| No | 49 (62.8) | 54 (68.4) | 56 (72.7) |
| **ECOG PS, *n* (%)** |  |  |  |
| 0 | 44 (56.4) | 45 (57.0) | 38 (49.4) |
| 1 | 34 (43.6) | 34 (43.0) | 39 (50.6) |
| **PR Positive^c^** |  |  |  |
| Yes | 60 (76.9) | 59 (74.7) | 62 (80.5) |
| No | 17 (21.8) | 19 (24.1) | 15 (19.5) |
| **Organ Sites, n** |  |  |  |
| 1 | 15 (19.2) | 21 (26.6) | 19 (24.7) |
| 2 | 29 (37.2) | 21 (26.6) | 19 (24.7) |
| ≥3 | 34 (43.6) | 37 (46.8) | 39 (50.6) |
| **ET for ABC^d^, n** |  |  |  |
| 1 | 21 (26.9) | 24 (30.4) | 26 (33.8) |
| 2 | 23 (29.5) | 26 (32.9) | 16 (20.8) |
| ≥3 | 16 (20.5) | 16 (20.3) | 15 (19.5) |
| **Chemotherapy regimens for ABC^e^, n** |  |  |  |
| 1 | 44 (56.4) | 38 (48.1) | 40 (51.9) |
| 2 | 29 (37.2) | 37 (46.8) | 33 (42.9) |
| 3 | 3 (3.8) | 0 | 0 |
| **Chemotherapy in any setting** |  |  |  |
| Taxanes | 68 (87.2) | 71 (89.9) | 66 (85.7) |
| Capecitabine | 30 (38.5) | 43 (54.4) | 27 (35.1) |

**Abbreviations**: A+T, abemaciclib 150 mg + tamoxifen; A-150, abemaciclib 150 mg; A-200, abemaciclib 200 mg + prophylactic loperamide; ABC, advanced breast cancer; ECOG PS, Eastern Cooperative Oncology Group performance status; ET, endocrine therapy; n, number of patients in category; PR, progesterone receptor

*Reproduced from Hamilton et al 2020 with permission from Elsevier*

^a^Percentage does not equal 100% because of rounding.

^b^Other category included American Indian, Alaskan native, Black, African American, and multiple.

^c^Two patients (one each in A+T arm and A-150 arm) had missing PR status.

^d^Previous fulvestrant in metastatic setting: 22 (28.2%) in A+T arm; 20 (25.3%) in A-150 arm; 18 (23.4%) in A-200 arm.

^e^Percentage does not equal 100% because 2 patients in A+T arm, 4 in A-150 arm, and 4 in A-200 arm had not received chemotherapy for ABC.**Supplemental Table 2 (online only). Summary of Drug Adjustments From Adverse Events: Number of Patients Having Treatment Doses Reduced, Omitted or Discontinued, Specific Number of Patients With 1, 2 Or ≥3 Abemaciclib Dose Reductions or Omissions and Most Common Adverse Events Listed^1^**

| **Variable** | **A+T Arm**  **(n=78)** | | **A-150 Arm**  **(n=79)** | **A-200 Arm**  **(n=77)** | |
| --- | --- | --- | --- | --- | --- |
|  | **Abemaciclib** | **Tamoxifen** | **Abemaciclib** | **Abemaciclib** | **Loperamide** |
| **Dose reductions (any)** | 22 (28.2) | 0 (0.0) | 27 (34.2) | 40 (51.9) | 6 (7.8) |
| **Dose reduction, reason** |  |  |  |  |  |
| Neutropenia | 9 (11.5) | – | 11 (13.9) | 8 10.4) | – |
| Diarrhea | 1 (1.3) | – | 4 (5.1) | 8 (10.4) | 1 (1.3) |
| Leukopenia | 2 (2.6) | – | 3 (3.8) | 5 (6.5) | – |
| Fatigue | 1 (1.3) | – | 1 (1.3) | 3 (3.9) | – |
| Muscular weakness | 1 (1.3) | – | 4 (5.1) | 0 (0.0) | – |
| **Dose omission (any)** | 37 (47.4) | 24 (30.8) | 43 (54.4) | 52 (67.5) | 22 (28.6) |
| **Dose omission, reason** |  |  |  |  |  |
| Neutropenia | 11 (14.1) | 2 (2.6) | 20 (25.3) | 19 (24.7) | 4 (5.2) |
| Diarrhea | 2 (2.6) | 0 | 6 (7.6) | 8 (10.4) | 1 (1.3) |
| Leukopenia | 3 (3.8) | 1 (1.3) | 4 (5.1) | 4 (5.2) | 1 (1.3) |
| Thrombocytopenia | 1 (1.3) | 0 (0.0) | 5 (6.3) | 5 (6.5) | 2 (2.6) |
| **Discontinuation, reason** | 7 (9.0) | | 8 (10.1) | 9 (11.7) | |
| ALT increased | 2 (2.6) | | 1 (1.3) | 1 (1.3) | |
| Neutropenia | 1 (1.3) | | 1 (1.3) | 2 (2.6) | |
| Cardiac arrest | 0 (0.0) | | 1 (1.3) | 1 (1.3) | |
| Thrombocytopenia | 0 (0.0) | | 1 (1.3) | 1 (1.3) | |

**Abbreviations:** A+T, abemaciclib 150 mg plus tamoxifen; A-150, abemaciclib 150 mg; A-200, abemaciclib 200 mg plus prophylactic loperamide, ALT, alanine aminotransferase; n, number of patients in category.

Data shows number of patients and percentage of all patients treated (safety population): n (%)

^1^Adverse events accounting for ≥5% of all abemaciclib adverse events listed

**Supplemental Table 3 (Online only): Overall Response Rate: Time to Response and Duration of Response (months)**

|  | **A+T Arm (n=27)** | | **A-150 Arm (n=19)** | | **A-200 Arm (n=26)** | |
| --- | --- | --- | --- | --- | --- | --- |
| **Responder population** | Month | 95% CI | Month | 95% CI | Month | 95% CI |
| Median time to response | 3.62 | 1.87-3.98 | 3.72 | 1.84-5.49 | 3.62 | 1.91-5.46 |
| Median duration of response | 7.40 | 3.75-9.27 | 8.40 | 3.75-14.83 | 8.45 | 5.56-18.44 |

**Abbreviations:** A+T, abemaciclib 150 mg plus tamoxifen; A-150, abemaciclib 150 mg; A-200, abemaciclib 200 mg plus prophylactic loperamide; n, number of patients in category.
